# Intravitreal Brolucizumab for Diabetic Macular Edema: Outcomes in Treatment-Naive and Anti-VEGF-Switched Eyes over 48 Weeks

**DOI:** 10.3390/jcm15135162

**Published:** 2026-07-02

**Authors:** Fumiaki Higashijima, Yugo Ota, Hikaru Jiromaru, Yoshinao Tamura, Masahiko Funatsu, Ren Aoki, Masanori Mikuni, Manami Ohta, Makiko Wakuta, Shinji Hirano, Kazuhiko Yamauchi, Kazuhiro Kimura

**Affiliations:** 1 Department of Ophthalmology, Yamaguchi University Graduate School of Medicine, Ube 755-8505, Yamaguchi, Japan; higashi@yamaguchi-u.ac.jp (F.H.); otayugo@yamaguchi-u.ac.jp (Y.O.); hjiro@yamaguchi-u.ac.jp (H.J.); yota1421@yamaguchi-u.ac.jp (Y.T.); orange9@yamaguchi-u.ac.jp (M.F.); renaoki@yamaguchi-u.ac.jp (R.A.); mikuni89@yamaguchi-u.ac.jp (M.M.); mohta@yamaguchi-u.ac.jp (M.O.); mwakut@yamaguchi-u.ac.jp (M.W.); s.hirano@yamaguchi-u.ac.jp (S.H.); 2 Yamaguchi Red Cross Hospital, Yamaguchi 753-0092, Yamaguchi, Japan; k.yama0420abba@r8.dion.ne.jp

**Keywords:** diabetic macular edema, brolucizumab, anti-VEGF, treatment-naive, switched, real-world, pro re nata, optical coherence tomography

## Abstract

**Background:** Brolucizumab has demonstrated efficacy for diabetic macular edema (DME) in pivotal clinical trials; however, comparative real-world data directly contrasting treatment-naive and anti-VEGF-switched eyes over a prolonged follow-up period remain limited. This case series evaluated 48-week functional, anatomical, and safety outcomes of intravitreal brolucizumab (IVBr) in treatment-naive and switched DME eyes in routine clinical practice. **Methods:** This retrospective, two-center case series included 21 eyes with center-involving DME treated with IVBr between May 2022 and April 2024. Eyes were classified into a treatment-naive group (*n* = 10), which included only eyes with no prior anti-VEGF treatment for DME, and a switched group (*n* = 11), which included eyes previously treated with other anti-VEGF agents but not achieving dry macula. Dry macula was defined as the absence of fovea-involving fluid on OCT. BCVA (logMAR), central retinal thickness (CRT), dry macula rate, the proportion of eyes gaining ≥2 lines of BCVA, number of injections, mean injection interval, and intraocular inflammation (IOI) were assessed at baseline and at 6, 12, 24, and 48 weeks. **Results:** At baseline, BCVA was 0.44 ± 0.27 in the treatment-naive group and 0.46 ± 0.28 in the switched group (*p* = 0.943), and CRT was 435.8 ± 150.1 μm and 461.8 ± 139.8 μm, respectively (*p* = 0.751). In the treatment-naive group, BCVA improved from 0.44 ± 0.27 to 0.24 ± 0.19 at week 48 (*p* = 0.023), and 5 eyes (50.0%) gained ≥2 lines. In the switched group, BCVA did not improve significantly (0.46 ± 0.28 to 0.41 ± 0.40; *p* = 0.461); 3 eyes (27.3%) gained ≥2 lines, and 1 eye (9.1%) lost ≥2 lines. CRT decreased significantly in treatment-naive eyes (435.8 ± 150.1 to 312.6 ± 106.2 μm, *p* = 0.020) and showed a non-significant reduction in switched eyes (461.8 ± 139.8 to 363.6 ± 127.4 μm, *p* = 0.067). Dry macula rates at week 48 were 40.0% and 27.3%, respectively (*p* = 0.659). Six switched eyes had prior intravitreal corticosteroid treatment for DME. One treatment-naive eye developed IOI, which resolved without permanent visual impairment. **Conclusions**: In this retrospective case series, IVBr was associated with anatomical improvement over 48 weeks, with clearer functional and anatomical responses in treatment-naive eyes than in switched eyes. However, the switched group was older and had a more heterogeneous treatment history, including prior intravitreal corticosteroid treatment. The findings should therefore be interpreted as exploratory.

## 1. Introduction

Diabetic macular edema (DME) is a leading cause of visual impairment in working-age adults worldwide, and intravitreal anti-vascular endothelial growth factor (VEGF) therapy is the established first-line treatment for center-involving disease [1,2]. Despite sustained anatomical improvements reported in pivotal randomized controlled trials, outcomes in routine clinical practice are often more modest, reflecting the challenges of reduced injection frequency, variable treatment adherence, and heterogeneous patient populations [3,4].

Brolucizumab is a single-chain antibody fragment that achieves high molar drug concentrations within the retina, enabling potent and sustained VEGF suppression. In the phase 3 KESTREL and KITE trials, brolucizumab demonstrated robust visual and anatomical improvements in DME at 52 weeks that were maintained through 100 weeks in many patients [5,6]. The KINGFISHER trial subsequently confirmed its efficacy in a broader DME population [7].

In clinical practice, brolucizumab may be used either as initial anti-VEGF therapy or after an insufficient response to other anti-VEGF agents. Whether its outcomes differ between these settings remains unclear, because most real-world studies have reported either treatment-naive eyes, switched eyes, or mixed cohorts without direct comparison [8,9,10]. Rübsam et al. directly compared brolucizumab outcomes in treatment-naive and recalcitrant DME eyes within a real-world cohort, but their study focused on comparison with aflibercept rather than on 48-week outcomes within a brolucizumab-treated cohort under PRN conditions [8].

It is also clinically relevant to know how visual and structural outcomes change under PRN-based management, which is common in routine care. PRN regimens have generally been associated with smaller visual gains than loading or treat-and-extend protocols in DME [11,12], but the influence of prior treatment status in this setting remains uncertain. We therefore evaluated 48-week outcomes of IVBr in treatment-naive and anti-VEGF-switched DME eyes treated in two centers.

## 2. Materials and Methods

### 2.1. Case Series Design and Ethics

This retrospective case series was conducted at Yamaguchi University Hospital and Yamaguchi Red Cross Hospital. This case series adhered to the tenets of the Declaration of Helsinki and was approved by the Institutional Review Board of Yamaguchi University Hospital (approval number: H2025-237; approval date: 25 March 2026). The requirement for written informed consent was waived because of the retrospective design; an opt-out approach was used where applicable.

### 2.2. Participants

Medical records of patients who initiated IVBr for center-involving DME between May 2022 and April 2024 were reviewed. Eligible eyes were followed for at least 48 weeks and had available BCVA and OCT data at baseline and follow-up visits. Eyes were excluded if macular edema was primarily attributable to another retinal disease, if active intraocular inflammation was present at baseline, or if severe media opacity or postoperative inflammation precluded reliable BCVA or OCT evaluation at baseline. A history of ocular surgery before IVBr initiation, including PPV or cataract surgery, was recorded as a baseline ocular characteristic rather than used as an exclusion criterion when baseline evaluation was reliable.

Eyes were classified into the treatment-naive group if they had not received any prior anti-VEGF therapy for DME. No eyes that restarted after a prolonged drug-free interval were included in the treatment-naive group. Eyes were classified into the switched group if they had previously received at least one anti-VEGF agent for DME and were switched to brolucizumab because fovea-involving fluid persisted or recurred on OCT despite prior treatment, and dry macula had not been achieved.

### 2.3. Treatment Protocol

The limited number of cases reflects the cautious use of brolucizumab in daily practice. During the observation period, brolucizumab was not used routinely for all DME eyes, mainly because of safety concerns regarding IOI and retinal vasculitis. It was selected when the treating physician considered that stronger anatomical drying was clinically needed. This approach is clinically understandable but inevitably introduces selection bias.

Some eyes underwent an initial loading phase followed by PRN treatment, whereas others were managed with a PRN-only regimen from treatment initiation. The loading phase was defined as three consecutive monthly IVBr injections followed by PRN retreatment. Loading was performed in 6 treatment-naive eyes and 3 switched eyes. Retreatment decisions were made at the discretion of the treating retinal specialist based on visual acuity, OCT findings, and disease activity.

### 2.4. Data Collection and Outcome Measures

Baseline variables included age, sex, duration of diabetes mellitus, hemoglobin A1c, diabetic retinopathy severity, history of panretinal photocoagulation, history of PPV, prior ocular surgery, prior anti-VEGF treatment history, and prior intravitreal corticosteroid treatment for DME. Because both eyes of some patients were included, age and sex were regarded as patient-level variables, whereas ocular variables were analyzed at the eye level.

Prior total anti-VEGF injections were recorded as 0 in the treatment-naive group and summarized descriptively in the switched group. In switched eyes, the number of prior anti-VEGF agents and the prior anti-VEGF switch count before IVBr were also recorded. Dry macula was defined as the absence of fovea-involving intraretinal and/or subretinal fluid on OCT; CRT alone was not used as the criterion for dry macula. The primary outcomes were BCVA (logMAR), CRT, and dry macula rate. The proportion of eyes gaining ≥2 lines (≥0.2 logMAR improvement) and losing ≥2 lines of BCVA was also recorded. Secondary outcomes were the number of IVBr injections, mean injection interval, and IOI during the 48-week follow-up.

### 2.5. Statistical Analysis

Continuous variables are presented as mean ± standard deviation (SD), and categorical variables as number (%). Baseline variables were compared using the Mann–Whitney U test or Fisher’s exact test. Within-group changes in BCVA and CRT from baseline to week 48 were assessed using the Wilcoxon signed-rank test, and longitudinal between-group comparisons were evaluated using generalized estimating equations (GEE). Dry macula rate was assessed descriptively using Fisher’s exact test at individual time points. The number of injections and mean injection interval were compared using the Mann–Whitney U test. Mean injection interval was calculated for eyes receiving two or more injections as the time from the first to the last injection divided by the number of injection intervals (total injections minus one) and was interpreted only as a descriptive treatment-burden metric because loading-plus-PRN and PRN-only regimens were both included.

Additional descriptive subgroup summaries were prepared according to loading status. Because of the small number of eyes in each subgroup, formal statistical testing was not performed for these subgroup summaries. A post hoc sensitivity analysis was also performed by excluding treatment-naive eyes with ocular surgery before IVBr initiation to assess the robustness of the main findings. A formal sample size calculation was not performed, given the retrospective design. A two-sided *p* value < 0.05 was considered statistically significant. All analyses were performed using JMP Pro (version 17; SAS Institute, Cary, NC, USA).

## 3. Results

### 3.1. Baseline Characteristics

Twenty-one eyes were included: 10 treatment-naive eyes and 11 switched eyes. The switched group was older than the treatment-naive group (74.4 ± 5.7 vs. 58.9 ± 13.2 years; *p* = 0.018). No eye in the treatment-naive group had previously received anti-VEGF therapy for DME.

In the switched group, the median number of prior anti-VEGF injections was 5 (range, 3–16), and the median number of prior anti-VEGF agents before IVBr was 1 (range, 1–3). The prior anti-VEGF switch count before IVBr was also summarized descriptively (median 0; range, 0–2). Six switched eyes (54.5%) had a history of intravitreal corticosteroid treatment for DME before IVBr initiation; no eyes received intravitreal corticosteroid therapy for DME during the 48-week IVBr follow-up period.

When an eye had undergone ocular surgery before IVBr, baseline BCVA and OCT were evaluated after surgery. Baseline BCVA and CRT did not differ between groups (both *p* > 0.05; Table 1).

### 3.2. Visual Outcomes

**Figure 1:**
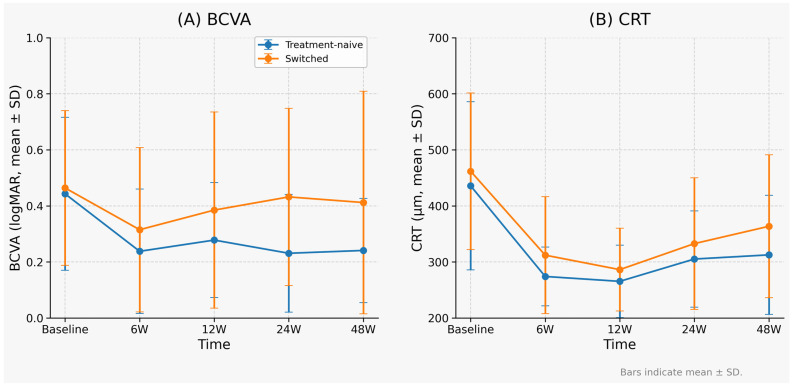
Longitudinal changes in primary outcomes over 48 weeks in treatment-naive and switched DME eyes treated with IVBr. (**A**) BCVA (logMAR): significant improvement was observed in the treatment-naive group (Wilcoxon signed-rank test, *p* = 0.023), but not in the switched group (*p* = 0.461). (**B**) CRT (μm): significant CRT reduction was observed in the treatment-naive group (*p* = 0.020), whereas the switched group showed a non-significant trend toward CRT reduction (*p* = 0.067). Error bars indicate SD. *p* values indicate within-group Wilcoxon signed-rank tests comparing baseline and week 48. GEE showed no significant between-group difference in either trajectory (both *p* > 0.05).

In treatment-naive eyes, BCVA improved from 0.44 ± 0.27 at baseline to 0.24 ± 0.19 at week 48 (*p* = 0.023). Five eyes (50.0%) gained ≥2 lines, and no eye lost ≥2 lines. In switched eyes, BCVA did not change significantly (0.46 ± 0.28 to 0.41 ± 0.40; *p* = 0.461). Three eyes (27.3%) gained ≥2 lines, and 1 eye (9.1%) lost ≥2 lines. GEE analysis showed no significant between-group difference in BCVA change over time (group-by-time interaction, *p* > 0.05) (Figure 1A).

### 3.3. Anatomical Outcomes

CRT decreased from 435.8 ± 150.1 to 312.6 ± 106.2 μm in treatment-naive eyes (*p* = 0.020). In switched eyes, CRT decreased from 461.8 ± 139.8 to 363.6 ± 127.4 μm, but this change did not reach statistical significance (*p* = 0.067). GEE analysis showed no significant between-group difference in CRT change over time (*p* > 0.05) (Figure 1B).

### 3.4. Dry Macula Rate and Injection Burden

**Figure 2:**
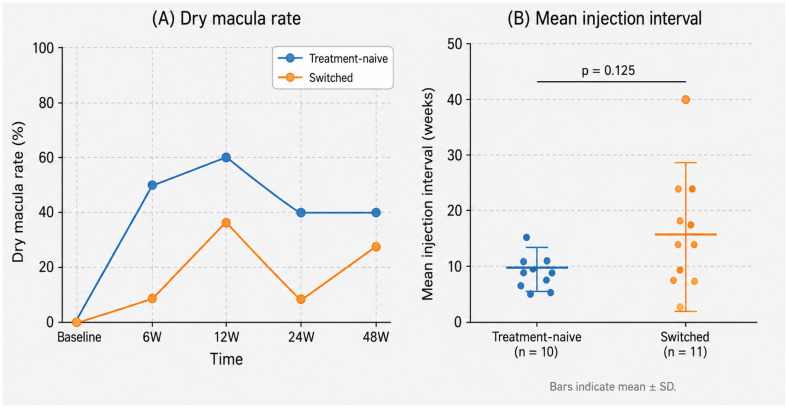
Longitudinal changes in secondary outcomes over 48 weeks. (**A**) Dry macula rate (%): rates at week 48 were 40.0% in the treatment-naive group and 27.3% in the switched group (*p* = 0.659). (**B**) Mean injection interval shown as individual data points with mean ± SD. Values were 9.96 ± 4.33 weeks in the treatment-naive group and 15.86 ± 12.89 weeks in the switched group (*p* = 0.125). Mean injection interval is shown only as a descriptive treatment-burden metric because loading-plus-PRN and PRN-only regimens were both included.

Dry macula rates at baseline, 6, 12, 24, and 48 weeks were 0%, 50.0%, 60.0%, 40.0%, and 40.0% in the treatment-naive group and 0%, 9.1%, 36.4%, 9.1%, and 27.3% in the switched group. A between-group difference was not statistically significant at week 6 (*p* = 0.063) or week 48 (*p* = 0.659) (Figure 2A). The dry macula rate in the switched group declined from 36.4% at week 12 to 9.1% at week 24 and partially recovered to 27.3% at week 48.

The number of injections was 3.90 ± 2.42 in the treatment-naive group and 4.18 ± 1.54 in the switched group (*p* = 0.560). Mean injection interval is shown only as a descriptive treatment-burden metric because loading-plus-PRN and PRN-only regimens were both included. The mean injection interval was 9.96 ± 4.33 weeks and 15.86 ± 12.89 weeks, respectively (*p* = 0.125) (Figure 2B).

### 3.5. Descriptive Analyses by Loading Status and Sensitivity Analysis

Descriptive outcomes stratified by loading status are presented in Supplementary Table S1. In a post hoc sensitivity analysis excluding treatment-naive eyes with ocular surgery before IVBr initiation, the direction of improvement in BCVA and CRT was preserved, although statistical significance was no longer reached because of the very small number of remaining eyes. BCVA changed from 0.48 ± 0.31 to 0.30 ± 0.19 (*p* = 0.156), and CRT changed from 396.0 ± 150.8 μm to 328.7 ± 122.2 μm (*p* = 0.156). This analysis supports cautious interpretation of the treatment-naive results without altering the overall exploratory interpretation of the case series.

### 3.6. Safety

One treatment-naive eye (10.0%) developed IOI 2 weeks after IVBr, with mild vitreous opacity and retinal arterial whitening, compatible with retinal vasculitis. The inflammation improved after sub-Tenon steroid injection without permanent visual impairment. This steroid injection was used for IOI management and was not part of DME treatment. No other serious adverse events occurred, and no case of retinal vascular occlusion or permanent visual loss was observed.

## 4. Discussion

This two-center case series compared 48-week outcomes of IVBr in treatment-naive and anti-VEGF-switched DME eyes. Functional and anatomical improvement was more apparent in treatment-naive eyes. In contrast, switched eyes showed a reduction in CRT without significant BCVA improvement. Because of the small sample size, treatment selection, baseline imbalance, and mixed treatment regimens, these findings should be viewed as exploratory.

In this case series, BCVA improved significantly in treatment-naïve eyes, whereas switched eyes showed limited BCVA improvement. Eyes switched from previous anti-VEGF therapy generally include cases with a longer treatment course or insufficient response to previous treatment. Therefore, their visual outcomes may be influenced by chronic edema, irreversible retinal damage, older age, prior corticosteroid exposure, and other unmeasured factors. Thus, prior anti-VEGF exposure should not be interpreted as a direct cause of poor visual recovery, but rather as a marker of chronic disease activity and treatment burden.

In the present series, CRT decreased significantly in treatment-naive eyes, whereas the reduction in switched eyes did not reach statistical significance. Although previous real-world studies have reported anatomical improvement after IVBr in switched or treatment-resistant DME eyes [8,9,10,13], treatment settings differed across studies, including structured loading, treat-and-extend treatment, short-term evaluation after a single injection, and larger non-strict PRN cohorts. Thus, the non-significant CRT reduction in our switched group may reflect the PRN-based regimen, limited sample size, and heterogeneity in the previous treatment course.

In switched eyes, CRT decreased numerically, but BCVA did not improve significantly. Previous studies have shown that anatomical improvement in DME does not always translate into visual recovery, particularly in chronic or recurrent cases, and OCT biomarkers such as ellipsoid zone disruption and DRIL have been associated with limited visual gain [14,15]. Recent real-world data also suggest that eyes requiring additional or alternative therapy may show limited functional recovery despite anatomical improvement [16,17]. Thus, the anatomical-functional discrepancy observed in our switched group may reflect chronic retinal structural changes and reduced treatment responsiveness rather than insufficient anatomical response alone.

In the switched group, the number of prior anti-VEGF injections and agents varied, and six eyes had prior intravitreal corticosteroid treatment. These treatment histories indicate that the switched group was not a uniform population. Because the number of switched eyes was small, the extent to which prior treatment burden influenced outcomes could not be assessed reliably.

Age is another important consideration. The switched group was significantly older than the treatment-naive group (74.4 ± 5.7 vs. 58.9 ± 13.2 years; *p* = 0.018). Older age may be associated with reduced retinal functional reserve and a higher likelihood of subclinical macular or neuroretinal damage. Because multivariable adjustment was not feasible, age remains an important confounder in interpreting the limited functional recovery in switched eyes.

Safety should also be interpreted cautiously. One eye developed IOI, which resolved without permanent visual impairment. Although this event was managed successfully, the cohort is too small to estimate the risk of IOI. This finding supports the need for careful patient selection and close monitoring after brolucizumab injection.

This case series has several limitations. First, the sample size was small, which limited statistical power, precluded multivariable analysis, and reduced generalizability. The case series was designed to describe outcomes in selected eyes treated with IVBr in routine practice, not to establish broad efficacy.

Second, some treatment-naive eyes had a history of ocular surgery before IVBr initiation. Sensitivity analysis excluding these eyes preserved the direction of BCVA and CRT improvement but no longer reached statistical significance, consistent with the very small sample size. This supports cautious interpretation of the treatment-naive results while preserving the exploratory value of the overall cohort.

Third, switched eyes were heterogeneous with respect to prior injection number, prior anti-VEGF agents, prior switch count, and prior intravitreal steroid exposure.

Fourth, OCT microstructural biomarkers such as ellipsoid zone integrity and DRIL were not systematically evaluated, limiting mechanistic insight into the anatomical-functional disparity.

Finally, the retrospective design precludes definitive causal conclusions.

## 5. Conclusions

In this retrospective case series, IVBr was associated with anatomical improvement over 48 weeks, with clearer functional and anatomical responses in treatment-naive eyes than in anti-VEGF-switched eyes. These findings should be interpreted cautiously because of the small sample size, treatment selection, mixed retreatment regimens, older age and heterogeneous treatment background in switched eyes, prior intravitreal corticosteroid exposure, and the non-significant CRT reduction in switched eyes. Thus, IVBr may be considered in selected DME eyes in which anatomical drying is clinically important, but careful patient selection and post-injection monitoring remain necessary, particularly in view of IOI. Further prospective studies using standardized treatment protocols and objective criteria for refractory DME are required.

## Figures and Tables

**Table 1:** Baseline characteristics.

Characteristic	Treatment-Naïve (10 Eyes; 8 Patients)	Switched (11 Eyes; 9 Patients)	*p* Value/Note
Age, years, mean (SD)	58.9 ± 13.2	74.4 ± 5.7	0.018
Male sex, patients, *n* (%)	4/8 (50.0%)	6/9 (66.7%)	0.637
Female sex, patients, *n* (%)	4/8 (50.0%)	3/9 (33.3%)	
Duration of diabetes, years, mean (SD)	13.2 ± 6.4	10.8 ± 5.1	1.000
HbA1c, %, mean (SD)	8.45 ± 1.59	7.50 ± 1.24	0.352
DR severity, NPDR, *n* (%)	3 (30.0%)	7 (63.6%)	0.198
DR severity, PDR, *n* (%)	7 (70.0%)	4 (36.4%)	
History of PRP, *n* (%)	6 (60.0%)	9 (81.8%)	0.361
History of PPV, *n* (%)	2 (20.0%)	0 (0.0%)	0.214
Prior intravitreal corticosteroid treatment before IVBr, *n* (%)	0 (0.0%)	6 (54.5%)	0.012
Prior total anti-VEGF injections, median (range)	0 (0–0)	5 (3–16)	<0.001
Number of prior anti-VEGF agents before IVBr, median (range)	0 (0–0)	1 (1–3)	<0.001
Prior anti-VEGF switch count before IVBr, median (range)	0 (0–0)	0 (0–2)	0.021
Baseline BCVA (logMAR), mean (SD)	0.44 ± 0.27	0.46 ± 0.28	0.943
Baseline CRT, μm, mean (SD)	435.8 ± 150.1	461.8 ± 139.8	0.751
Prior anti-VEGF agents in switched group	None	—	
Aflibercept, *n* (%)	0	11 (100.0%)	—
Ranibizumab, *n* (%)	0	5 (45.5%)	—
Faricimab, *n* (%)	0	3 (27.3%)	—

*p* values for continuous variables were calculated using the Mann–Whitney U test, and *p* values for categorical variables were calculated using Fisher’s exact test. Because both eyes from some patients were included, age and sex were treated as patient-level variables, whereas ocular characteristics were summarized at the eye level. Diabetes duration and HbA1c were analyzed using available-case data. Prior anti-VEGF switch count before IVBr was summarized descriptively; the switch to brolucizumab itself was not included. DR, diabetic retinopathy; NPDR, non-proliferative diabetic retinopathy; PDR, proliferative diabetic retinopathy; PRP, panretinal photocoagulation; PPV, pars plana vitrectomy; BCVA, best-corrected visual acuity; CRT, central retinal thickness; SD, standard deviation.

## Data Availability

The data presented in this case series are not publicly available due to ethical and privacy restrictions. De-identified data may be available from the corresponding author upon reasonable request and with institutional approval.

## Supplementary Materials

The following supporting information can be downloaded at: https://www.mdpi.com/article/10.3390/jcm15135162/s1. Supplementary Table S1: Descriptive subgroup summary stratified by loading status. Formal statistical testing was not performed because of the small number of eyes in each subgroup.
