# Supplementary material for: Intravitreal Brolucizumab for Diabetic Macular Edema: Outcomes in Treatment-Naive and Anti-VEGF-Switched Eyes over 48 Weeks
Source: J Clin Med. 2026 Jul 2;15(13):5162. doi: 10.3390/jcm15135162 (PMC13362911; doi:10.3390/jcm15135162)
Supplement: Supplementary file 1 [file jcm-15-05162-s001.zip › jcm-4334441-supplementary.pdf]

Supplementary Table S1. Descriptive subgroup summary stratified by loading status. Formal statistical testing was not performed because of the small number of eyes in each subgroup.

Group	Loading status	n	Baseline BCVA mean	Baseline BCVA SD	Week 48 BCVA mean	Week 48 BCVA SD	ΔBCVA mean	ΔBCVA SD	Baseline CRT mean	Baseline CRT SD	Week 48 CRT mean	Week 48 CRT SD	ΔCRT mean	ΔCRT SD
Naïve	PRN-only/no loading	4	0.565	0.261	0.26	0.215	-0.305	0.152	347	86.4	313.2	121.6	-33.8	57.1
Naïve	Loading	6	0.362	0.271	0.228	0.184	-0.133	0.207	495	159.9	312.2	107.0	-182.8	168.8
Switched	PRN-only/no loading	8	0.51	0.267	0.445	0.434	-0.065	0.344	483.6	157.5	360	124.0	-123.6	138.2
Switched	Loading	3	0.34	0.317	0.323	0.337	-0.017	0.029	403.7	62.4	373.3	164.6	-30.3	116.0
